# Dataset for analysing the relationships among economic growth, fossil fuel and non-fossil fuel consumption

**DOI:** 10.1016/j.dib.2016.11.076

**Published:** 2016-11-26

**Authors:** John Asafu-Adjaye, Dominic Byrne, Maximiliano Alvarez

**Affiliations:** School of Economics, University of Queensland, Brisbane QLD 4072, Australia

**Keywords:** Economic growth, Fossil fuel consumption, Non-fossil fuel consumption, Physical capital, Human capital

## Abstract

The data presented in this article are related to the research article entitled ‘Economic Growth, Fossil Fuel and Non-Fossil Consumption: A Pooled Mean Group Analysis using Proxies for Capital’ (J. Asafu-Adjaye, D. Byrne, M. Alvarez, 2016) [1]. This article describes data modified from three publicly available data sources: the World Bank׳s World Development Indicators (http://databank.worldbank.org/data/reports.aspx?source=world-development-indicators), the U.S. Energy Information Administration׳s International Energy Statistics (http://www.eia.gov/cfapps/ipdbproject/IEDIndex3.cfm?tid=44&pid=44&aid=2) and the Barro-Lee Educational Attainment Dataset (http://www.barrolee.com). These data can be used to examine the relationships between economic growth and different forms of energy consumption.

The dataset is made publicly available to promote further analyses.

**Specifications Table**

**Table:** 

**Subject area**	Economics
**More specific subject area**	Energy Economics
**Type of data**	Links
**How data was acquired**	Publicly available online
**Data format**	Raw, Analysed
**Experimental factors**	Publicly available data sources
**Experimental features**	The data are calculated based on published data
**Data source location**	Global data
**Data accessibility**	The data are available with this article. and are publicly available from
	The World Development Indicators is available at: http://databank.worldbank.org/data/reports.aspx?source=world-development-indicators
	The International Energy Statistics is available at: http://www.eia.gov/cfapps/ipdbproject/IEDIndex3.cfm?tid=44&pid=44&aid=2
	The Barro-Lee Educational Attainment Dataset is available at: http://www.barrolee.com

**Value of the data**

• The data are useful for analysing the relationship between economic growth and different forms of energy consumption.
• The data are publicly available, but are dispersed within several different sources.
• The data are collected for 53 countries with varying levels of economic development.
• Other researchers may find the data useful for different types of analysis in areas such as environmental and development economics.

## 1 Data

The data comprises real gross domestic product (GDP) and physical capital from the World Bank, fossil fuel and non-fossil fuel consumption from the U.S. Energy Information Administration and human capital from the Barro-Lee Educational Attainment Dataset for 53 countries over the period 1990–2012. Real GDP and physical capital stock are in millions of 2005 US dollars, fossil and non-fossil fuel consumption are both measured in trillions of British thermal units and human capital is in millions.

## 2 Experimental design, materials and methods

The data on real GDP were obtained from the World Bank׳s World Development Indicators (WDI) [2]. The data to construct fossil fuel consumption were extracted from the database of the U.S. Energy Information Administration (EIA) database [3]. Fossil fuel consumption was calculated as the sum of petroleum, coal and natural gas consumption. The EIA does not publish data on non-fossil fuels, so this was derived as total energy consumption (obtained from the database) less fossil fuel consumption.

The data on physical capital were constructed by accumulating real gross fixed capital formation (obtained from the WDI) assuming a constant depreciation rate using the perpetual inventory method [4] and a depreciation rate of 4%.

The data on human capital (*H*_*it*_) were constructed as follows:

$$H_{it}=L_{it}h_{it}$$

where *L*_*it*_=total labour force (millions of people, obtained from the WDI)

$$h_{it}=e^{rs_{it}}$$

where *s*_*it*_=average years of schooling (obtained from [5]) *r*=return to education, set at 10% following [6].

All the data were converted into logarithms for the purposes of the analysis [1].

## References

[1] J.Asafu-Adjaye, D.Byrne, M.Alvarez, Economic growth, fossil fuel and non-fossil consumption: A pooled mean group analysis using proxies for capital. Energy Econ. 〈http://dx.doi.org/10.1016/j.eneco.2016.10.016〉

[2] World Bank, World development indicators, Online version, 〈http://databank.worldbank.org/data/reports.aspx?Source=world-development-indicators〉, 2015.

[3] U.S Energy Information Administration (EIA), International Energy Statistics, 〈http://www.eia.gov/cfapps/ipdbproject/IEDIndex3.cfm?Tid=44&pid=44&aid=2〉

[4] Berlemann M., Wesselhöft J.-E. (2014). Estimating aggregate capital stocks using the perpetual inventory method: a survey of previous implementations and new empirical evidence for 103 countries. Rev. Econ..

[5] Barro R., Lee J.-W. (2015). A new data set of educational attainment in the world, 1950–2010. J. Dev. Econ..

[6] Pritchett L. (2001). Where has all the education gone?. World Bank Econ. Rev..

